# Effect of a 12-Week Summer Break on School Day Physical Activity and Health-Related Fitness in Low-Income Children from CSPAP Schools

**DOI:** 10.1155/2017/9760817

**Published:** 2017-03-09

**Authors:** You Fu, Timothy A. Brusseau, James C. Hannon, Ryan D. Burns

**Affiliations:** ^1^University of Nevada, 1664 North Virginia Street, Reno, NV 89557, USA; ^2^University of Utah, 250 S. 1850 E., Salt Lake City, UT 84112, USA; ^3^West Virginia University, 375 Birch St., Morgantown, WV 26505, USA

## Abstract

*Background*. The purpose of this study was to examine the effect of a 12-week summer break on school day physical activity and health-related fitness (HRF) in children from schools receiving a Comprehensive School Physical Activity Program (CSPAP).* Methods*. Participants were school-aged children (*N* = 1,232; 624 girls and 608 boys; mean age = 9.5 ± 1.8 years) recruited from three low-income schools receiving a CSPAP. Physical activity and HRF levels were collected during the end of spring semester 2015 and again during the beginning of fall semester 2015. Physical activity was assessed using the Yamax DigiWalker CW600 pedometer. HRF measures consisted of body mass index (BMI) and the Progressive Aerobic Cardiovascular Endurance Run (PACER).* Results*. Results from a doubly MANCOVA analysis indicated that pedometer step counts decreased from 4,929 steps in the spring to 4,445 steps in the fall (mean difference = 484 steps; *P* < 0.001; Cohen's* d* = 0.30) and PACER laps decreased from 31.2 laps in the spring to 25.8 laps in the fall (mean difference = 5.4 laps; *P* < 0.001; Cohen's* d* = 0.33).* Conclusions*. Children from schools receiving a CSPAP intervention had lower levels of school day physical activity and cardiorespiratory endurance following a 12-week summer break.

## 1. Introduction

Despite the numerous benefits of meeting recommended levels of physical activity [1–3], a majority of children are not meeting 60 minutes of moderate-to-vigorous physical activity (MVPA) per day [3, 4]. Recently, the Institutes of Medicine has recommended at least one-half of the recommended levels of MVPA should be achieved during school hours [5]. Because the majority of school day is spent in sedentary behaviors, achieving at least 30 minutes of MVPA during school hours presents a challenge. The Centers for Disease Control and Prevention has recommended that schools adopt Comprehensive School Physical Activity Programming (CSPAP) to facilitate achievement of school day MVPA [6]. CSPAP is a multicomponent approach in which schools use all available resources to improve the physical activity behaviors of children [6]. Multicomponent school-based interventions have been shown to be effective in promoting school day physical activity during the school year [7, 8]; however the long-term sustainability and efficacy of this behavioral model are questionable.

Although the goal of CSPAP is to improve MVPA levels in children, it is the physiological trait of having healthy levels of body composition and/or higher levels of cardiorespiratory endurance that has a stronger protective effect on developing cardiometabolic disease risk factors in the pediatric population [9, 10]. Over the past couple of decades, there has been an emphasis placed on improving health via increasing health-related fitness (HRF) levels in children [11]. The five domains of HRF include body composition, cardiorespiratory endurance, muscular strength and endurance, and flexibility [11]. Of these five domains, body composition and cardiorespiratory endurance have the strongest links to health outcomes [12, 13]. Therefore, studies aiming to examine the efficacy of school-based physical activity interventions like CSPAP should also analyze change in HRF levels.

All children can benefit from optimal levels of physical activity and HRF; however low-income children may especially benefit [14, 15]. Low-income and/or disadvantaged children have relatively fewer opportunities to participate in free-living physical activity during the day, have poor built-in environments conductive for physical activity participation, and, if of an ethnic minority, have a greater prevalence of unfavorable cardiometabolic biomarkers compared to non-Hispanic Caucasian children and/or children of a higher socioeconomic classification [16–18]. Indeed, it has been shown that low socioeconomic status children display lower levels of MVPA compared to higher socioeconomic status children [19]. Therefore, school-based interventions like CSPAP have the potential to greatly benefit this specific pediatric population.

Despite the potential benefits of programs like CSPAP, its long-term efficacy has not been established. A time period that may attenuate the potential benefits gained from a CSPAP intervention is during summer break. Although most studies show that physical activity behaviors are the highest during the summer months in children, some studies have indicated that physical activity is compromised during the summer, possibly because of weather patterns displaying outside temperatures that are too hot and uncomfortable [20], typical to summer temperatures recorded in the geographic regions of the South and Southwestern US [21]. Declines in physical activity during the summer months in these geographic regions may contribute to decreases in HRF levels. This phenomenon may be more prevalent in children from low-income families because of the factors that were stated previously. Thus, the benefits of the CSPAP on children may have the potential to be lost during a long break between school sessions. Despite this possibility, no research has examined the influence that a summer break has on physical activity and HRF levels in low-income children who are enrolled in schools receiving CSPAP. Therefore, the purpose of this study was to examine the effect of a 12-week summer break on physical activity and HRF in children from schools receiving a CSPAP intervention. It was hypothesized that the children's physical activity and HRF levels will decrease from the end of spring semester to the beginning of fall semester.

## 2. Methods

### 2.1. Participants

Participants were a convenience sample of 1,232 school-aged children (624 girls and 608 boys; mean  age = 9.5 ± 1.8 years) recruited from three low-income elementary schools receiving government financial assistance (i.e., “Title I Schools”) from the Mountain West Region of the US. The schools were receiving a CSPAP intervention during the time of data collection. The CSPAP at each of the three schools was for three years. The data used in this study consisted of measures collected at the end of the first CSPAP year and the beginning of the second CSPAP year. Children were recruited from the 1st to 6th grades. Approximately 60.6% of the sample was of Hispanic/Latino ethnicity, 13.7% was Pacific Islander, 10.0% was Caucasian, 7.8% was African American, 3.5% was Asian, and approximately 4.0% was classified as other. Written assent was obtained from the students and consent was obtained from the parents prior to data collection. The University Institutional Review Board approved the protocols employed in this study.

### 2.2. Instrumentation

#### 2.2.1. Physical Activity

Physical activity was measured using Yamax DigiWalker CW600 pedometers (Tokyo, Japan). Each student in the sample (*N* = 1,232) wore a pedometer for one school week. The pedometers were worn for 5 school days (Monday through Friday) between the hours of 8 a.m. and 3 p.m. on the right hip at the level of the iliac crest in line with the right knee. Classroom teachers, physical educators, and members of the research team ensured that the devices were worn during the entirety of the school day.

The pedometers included a seven-day memory that was used to record steps each day of the school week. Yamax DigiWalker models have been shown to be a valid measure for physical activity in children [22]. The Yamax DigiWalker pedometers have also been shown to provide a reliable estimate of step counts in elementary school-aged children [23]. Participants were included in the analysis if they had recorded data for at least 3 valid days of the school week, to ensure that the devices were worn for the majority of the school week, and had valid data at both spring and fall time-points (1,232/1,260; 97.7%).

#### 2.2.2. Health-Related Fitness

Body mass index (BMI) was calculated using standard procedures taking a student's weight in kilograms divided by the square or his or her height in meters. Height was measured to the nearest 0.01 meters using a portable stadiometer (Seca 213; Hanover, MD, USA) and weight was measured to the nearest 0.1 kilograms using a portable medical scale (BD-590; Tokyo, Japan). Height and weight were collected in a private room during each student's physical education class.

Cardiorespiratory endurance was measured using the 20-meter Progressive Aerobic Cardiovascular Endurance Run (PACER), administered during each student's physical education class. The PACER was conducted on a marked gymnasium floor with background music provided by a compact disc. Each student was instructed to run from one floor marker to another floor marker across a 20-meter distance within an allotted time frame. The allotted time given to reach the specified distance incrementally shortened as the test progressed. If the student twice failed to reach the other floor marker, the test was terminated [24]. The final score was recorded in laps.

### 2.3. Procedures

Data were collected at the end of spring semester, 1–3 weeks before the last day of school, and again at the beginning of fall semester, approximately 1–3 weeks after the first day of class. Pedometers were handed out to the homeroom teachers on a Monday morning and then were given to each student. Each pedometer had an identification number and was given to the student with the corresponding identifier. Before handing the pedometers to each homeroom teacher, each pedometers' accuracy in measuring steps was checked using the “shake” test, using the procedures outlined by Vincent and Sidman [25]. Students put on the pedometers using the aforementioned procedures at approximately 8 a.m. every morning and took off the pedometers at 3 p.m. in the afternoon. The pedometers were not taken off during any time during the school day. Pedometers were collected from the homeroom teachers on Friday and the data were then entered into an Excel spreadsheet.

HRF measures were collected during physical education. Students entered a private screening area to have their height and weight collected. The PACER was administered in sex-specific groups of approximately 8–12 children per group. The PACER was administered indoors at each of the three schools during both time-points. One trained research assistant collected all anthropometric measures and one trained research assistant collected PACER measures at each school to maintain testing consistency.

### 2.4. Statistical Analysis

Data were screened for outliers using *z*-scores and box plots and checked for Gaussian distributions using *k*-density plots. A 6 × 2 × 2 doubly Multivariate Analysis of Covariance (MANCOVA) was employed to examine the effect of grade level (1st–6th grade), sex (girl, boy), and time (spring and fall) on average school day step counts and PACER laps, adjusting for school and classroom level clustering. Statistically significant multivariate effects were followed by separate univariate Analysis of Covariance (ANCOVA) tests with a Bonferroni alpha level adjustment to protect against potentially inflated Type I error. If statistically significant grade main effect was found, a Bonferroni post hoc test with further alpha level adjustment was used. Cohen's delta (*d*) determined the effect size and practical significance of each pairwise comparison. Effect sizes were classified as small if *d* ≤ 0.2, medium if *d*≅0.5, and large if *d* ≥ 0.8 [26]. The MANOVA assumption of equality of population covariance matrices was checked using Box's *M* test. Alpha level was originally set at *P* ≤ 0.05 and all analyses were carried out using SPSS v21.0 statistical software package (Armonk, NY, USA).

## 3. Results

**Figure 1:**
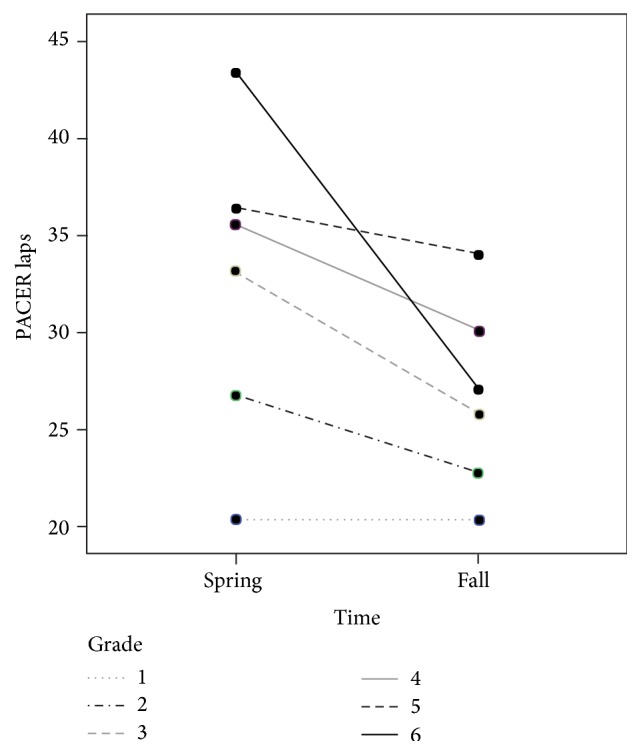
Changes in PACER laps between spring and fall time-points by grade level.

The descriptive statistics for the total sample and within each sex group are reported in Table 1 at both spring and fall time-points. There were statistically significant multivariate main effects for grade (Wilks' Λ = 0.92; *F* = 6.3;  *P* < 0.001), sex (Wilks' Λ = 0.99; *F* = 3.9;  *P* = 0.008), and time (Wilks' Λ = 0.98; *F* = 5.8;  *P* < 0.001) and a statistically significant multivariate grade × time interaction (Wilks' Λ = 0.97; *F* = 5.5;  *P* < 0.001). Data in Table 1 displays the differences between time-points for health-related fitness (BMI and PACER laps) and school day step counts, respectively. Follow-up ANOVA tests revealed that the mean pedometer step counts decreased from 4,929 steps in the spring to 4,445 steps in the fall (mean difference = 484 steps; *P* < 0.001; Cohen's* d* = 0.30) and mean PACER laps decreased from 31.2 laps in the spring to 25.8 laps in the fall (mean difference = 5.4 laps; *P* < 0.001; Cohen's* d* = 0.33). Both mean differences represented a small-to-medium sized effect. Follow-up ANCOVA tests also revealed a statistically significant univariate grade × time interaction for PACER laps (*F* = 8.2;  *P* < 0.001).  Figure 1 is a line plot displaying the change in PACER laps between spring and fall time-points by grade level. Children in the sixth grade showed significantly greater decreases between spring and fall time-points in PACER laps compared to all other grade levels (*P* < 0.001) and children in second through sixth grades displayed significantly greater decreases in PACER laps compared to children from the first grade. In particular, the average BMI for girls (*n* = 624) was 17.8 (SD = 4.6), and average BMI for boys (*n* = 608) was 18.8 (SD = 5.4). According to CDC's (The Centers for Disease Control and Prevention) BMI-for-age growth charts [27], both girls and boys in this study are averagely classified into the normal weight category. There were no mean differences between the spring and fall time-points on BMI (*P* > 0.01).

## 4. Discussion

The purpose of this study was to examine the effect of a 12-week summer break on physical activity and HRF in low-income children who were enrolled in three CSPAP schools. The results indicated that step counts and PACER laps were lower at the beginning of the fall semester compared to the previous spring semester, prior to a 12-week summer break. This study provides empirical evidence that any benefits from a CSPAP intervention may be lost when children are out of school. Because data were collected on low-income children, the results do not generalize to higher socioeconomic pediatric populations. Strategies must be devised from personnel working within a CSPAP model to attenuate the declines in physical activity and HRF that may occur during a long break in between school sessions.

An explanation of the CSPAP intervention is needed to communicate the potential benefits these school-based programs may manifest across a school year. CSPAP's primary focus was to provide training and assistance to improve the quality of physical education at each of the three schools. Specifically, monthly in-service opportunities and teacher training were provided to ensure that physical education met national standards, was student-centered, and was developmentally appropriate. The goal was set for teachers to maximize physical activity opportunities through greater student engagement, improved lesson planning, and decreased management and waiting time. Physical education was taught one day per week for 50 minutes and Dynamic Physical Education for Elementary School Children curriculum was employed [28].

During the CSPAP intervention, classroom teachers were asked to implement at least one and encouraged to regularly attempt three-minute activity breaks throughout the day using general activity breaks (including Energizers) or the “TAKE10!” program [29]. Examples of physical activity breaks in the classroom included a stretching or relaxation break, walking around the classroom or hallway, jumping with an invisible jump rope, doing squats, push-ups, sit-ups, and/or passing a ball around the classroom.

In addition to improving the quality of physical education, CSPAP offered physical activity opportunities throughout the school day during recess. Recesses were led by a Physical Activity Leader (PAL) and offered a significant number of opportunities for children to engage in free play or semistructured physical activity while also allowing them to apply the skills learned during their physical education lessons. Each school offered a 15-minute recess immediately following lunch as well as a 15-minute afternoon recess (the length and frequency of recess did not change throughout the school year).

CSPAP is becoming a popular model for increasing children's physical activity levels during the school day. Using the aforementioned behavioral model, in this sample of low-income children, CSPAP increased average steps per day by approximately 600 steps and increased time in MVPA by approximately 4.0 minutes per day between the beginning and end of the school year (in review). In addition, cardiorespiratory levels increased, on average, by approximately 6.5 PACER laps (in review). Both of these increases represented a small-to-medium sized effect (*d* ≈ 0.4; in review). However, as the results from this study have indicated, pedometer step counts decreased by approximately 500 steps per school day and PACER laps decreased by 5.4 laps. Therefore, summer breaks may impose a threat to the long-term efficacy of CSPAP programming in low-income children.

The lower step counts recorded during fall semester compared to spring semester may not totally reflect lower physical activity behaviors during the summer months. The lower step counts displayed in the fall semester in this sample may have reflected the novelty of the school year for both students and staff and the partial implementation of the CSPAP program itself, as teachers may not have fully implemented physical activity breaks, PE lessons may not have commenced, and the PALs at each of the three schools may not have yet devised plans to provide students with physical activity opportunities at recess.

Although the summer months have been shown to be a time period where physical activity levels tend to be higher in children [30, 31], some well-designed longitudinal studies have showed that physical activity may actually be compromised during the summer, especially in girls [20], in climates where outdoor temperatures are too hot and uncomfortable for children to consistently participate in active play. Previous study has also suggested that there are decreases in MVPA and increases in sedentary behaviors as school students grow up [32], and these unfavorable trends have been more drastic in girls compared with boys. This explains that girls demonstrated an overall lower PA level than boys in this study. Data in this study were collected from a state from the Mountain West Region of the US, where temperatures are often in the range of 90 to 100 degrees Fahrenheit during the majority of a summer day. Therefore, it is possible that hot and uncomfortable summer temperatures may compromise the physical activity behaviors of low-income children in certain regions of the US. The phenomenon, in addition to the barriers that low-income children face to achieve 60 minutes of MVPA per day, makes it theoretically logical that summer breaks would be significantly detrimental to optimize physical activity and HRF in this pediatric population. Although no mean BMI differences between the spring and fall time-points were found, both girls and boys demonstrated slight increase on their BMI. A plausible explanation is that the children gained their weights during the summer days due to the lack of the organized physical education classes, which also echoes the facts that participants' PA levels were decreased during the summer time.

The lower recorded step counts in the fall may reflect decreases in ambulatory physical activity behaviors and thus may have affected cardiorespiratory endurance levels. Cardiorespiratory endurance is an important component to HRF in children and can distinguish, with a reasonable amount of accuracy, children who have unfavorable cardiometabolic biomarkers from healthy children [33]. The average PACER score at the fall time-point was approximately 5 laps fewer compared to the spring time-point. The decrease in cardiorespiratory endurance may be attributable to decreases in ambulatory physical activity during the summer months. Interestingly, first graders in this sample did not experience any mean decrease in PACER laps, while the sixth graders' PACER laps drastically decreased between the two time-points. At the commencement of adolescence, children's MVPA decreases significantly and this trend tracks throughout the developmental years and into young adulthood [34, 35]. Decreases in MVPA may be accompanied by decreases in cardiorespiratory endurance levels [35, 36]. Sixth graders in this sample had nearly a 15-lap decrease in PACER laps from spring to fall time-points. If not addressed, low levels of cardiorespiratory endurance may track through adolescence and further influence health risk [37].

The results from this study manifest important practical implications. Even though CSPAP has the potential to increase physical activity and HRF levels in children, breaks in CSPAP may lead to a decline in these constructs and ultimately attenuate any benefits it may have accumulated across a school year. Practitioners and teachers working within the CSPAP model need to be aware of these potential declines after a break from CSPAP programming. Although it is difficult to follow up students during the summer months, devising a physical activity or HRF summer plan for each student within CSPAP schools may facilitate the continuation of CSPAP principles throughout the summer months. Although adherence to the principles is not guaranteed, especially in younger children, it still may partially attenuate potential decreases in physical activity and HRF during long school breaks, especially in those children who do not have many physical activity opportunities during the summer. Physical activity experts and physical education teachers may consider other strategies. Because each school has its own unique characteristics in student composition, geographical area, and proximately to physical activity promoting facilities (e.g., family centers and playgrounds), derivation of physical activity strategies during the summer months needs to be constructed to meet each school's and most specifically to meet each child's needs. It will be advantageous for CSPAP personnel to address the aforementioned considerations prior to the end of the school year.

There are limitations to this study that must be considered before any generalizations can be made. First, the majority of the sample consisted of low-income children who were of an ethnic minority; therefore the external validity of the results is questionable if generalized to other populations of children with different ethnic and/or socioeconomic representation. Also, the geographical regions may have significantly influenced the results; therefore the external validity of the results is highly questionable if generalized to other geographical regions, especially regions characterized by summers with relatively cooler average temperatures. Also, there was no control group to compare the CSPAP schools with schools that do not employ CSPAP. The internal validity of the results would be stronger if CSPAP schools were compared to non-CSPAP schools. Finally, measurement of physical activity consisted of using pedometers to record step counts. Pedometers do not capture the intensity of ambulatory physical activity and only capture lower-body movements; therefore the construct validity of this instrument is questionable if one were to generalize the results to whole body physical activity of varying intensities. However, because of the large sample size and time constraints for collecting and analyzing the data, pedometers were considered to be the most efficient objective instrument for population-based physical activity surveillance in school settings.

In conclusion, low-income children from CSPAP schools displayed fewer step counts and PACER laps in the fall compared to the previous spring. For cardiorespiratory endurance, older elementary school-aged children showed greater decreases compared to children in the first grade. The results provide empirical evidence suggesting that the improvements accumulated over one year of CSPAP may be partially or fully eliminated after long breaks in school sessions in low-income children. Practitioners and teachers that are involved in CSPAP programs need to be aware of the potential declines in physical activity and HRF levels that may occur after a summer break in low-income children. Strategies must be devised late in the school year to help attenuate these potential declines in physical activity and HRF. Although CSPAP shows promise to improve the health of children, long breaks in between school sessions threaten the long-term efficacy of such programming.

## Figures and Tables

**Table 1:** Mean health-related fitness and pedometer steps for sex groups at spring and fall time-points.

	Girls (*n* = 624)		Boys (*n* = 608)	
	Spring	Fall	Spring	Fall
BMI^a^	17.4 ± 4.0	17.8 ± 5.3	18.1 ± 6.4	19.6 ± 4.4
PACER^b^ laps	28.0 ± 16.5	23.5 ± 14.1^*∗*^	32.9 ± 19.0	28.4 ± 18.4^*∗*^
Steps per school day	4,796 ± 1873	4,319 ± 1572^*∗*^	5,043 ± 2045	4,586 ± 1852^*∗*^

*Note*. ^a^BMI stands for body mass index; ^b^PACER stands for the Progressive Aerobic Cardiovascular Endurance Run; ^*∗*^statistically significant difference compared to the spring time-point, *P* < 0.01.
